# Evaluation of the population dynamics of microalgae isolated from the state of Chiapas, Mexico with respect to the nutritional quality of water

**DOI:** 10.3897/BDJ.6.e28496

**Published:** 2018-09-26

**Authors:** Yazmin Sánchez Roque, Yolanda del Carmen Pérez-Luna, Joel Moreira Acosta, Neín Farrera Vázquez, Roberto Berrones Hernández, Sergio Saldaña Trinidad, Joseph Sebastian Pathiyamattom

**Affiliations:** 1 Universidad Politécnica de Chiapas, Suchiapa, Mexico Universidad Politécnica de Chiapas Suchiapa Mexico; 2 Universidad de Ciencias y Artes de Chiapas, Tuxtla Gutiérrez, Mexico Universidad de Ciencias y Artes de Chiapas Tuxtla Gutiérrez Mexico; 3 Universidad Autónoma de México, Cuernavaca, Mexico Universidad Autónoma de México Cuernavaca Mexico

**Keywords:** Hydrographic areas, isolation, microalgae

## Abstract

As Chiapas state, México, counts on an extensive hydrography with diverse nutrimental and climatic characteristics, it therefore allows isolating and identifying microalgae with bioenergetics potential.

For this purpose, samples from 8 locations were collected, corresponding to 6 rivers, a wastewater and a springwater. The isolation of microalgae was developed for 4 weeks with 12:12 light/dark cycles. We demonstrated that the most efficient means for the isolation of microalgae of the hydrographic areas evaluated was the medium BG11 with 80.53% effectiveness. Of the microalgal consortium identified, 90% are composed of microalgae belonging to the class Chlorophycear. It was shown that another factor favouring the richness of morphotypes identified in the Santo Domingo River is associated with adequate concentrations of macroelements such as nitrates, nitrites, ammonium, phosphorus, sodium, potassium, magnesium and calcium at concentrations of 0.03 mg/l, 0.0006 mg/l, 0.08 mg/l, 0.03 mg/l, 62.93 mg/l, 5.46 mg/l, 34.52 mg/l and 48.78 mg/l respectively and microelements such as copper, zinc, iron, andmanganese at concentrations less than 0.2 mg/l in all microelements. The identified morphotypes, according to literature, have lipid contents ranging from 2 to 90%; this is of biotechnological importance for the production of biodiesel.

## Introduction

As the human population increases, world energy demand and dependence on fossil fuels have continued to increase. As a result, global carbon emissions, including greenhouse gases, have increased to contribute to global warming, so the need to move towards sustainable alternatives to fossil fuel use is not only necessary to addressClimate change, but also to address the depletion of world energies (Griffiths and Harrison 2009).

Biofuels generated from algae in particular have been identified as an exceptional source of carbon and renewable energy (Schenk et al. 2008, Hussain et al. 2017, Clarens et al. 2010). Its high photosynthetic efficiency, biomass production and the ability to accumulate relatively large amounts of triacylglycerides (TAGs) for the conversion of fatty acid methyl esters (FAME) have made them a desirable alternative for the production of biofuels. They are known as microalgae to the set of unicellular eukaryotic and prokaryotic microorganisms (cyanobacteria) that synthesize a large amount of chlorophyll-a and other pigments that define its green colouration, allow them to perform photosynthesis and act as primary producers in the generation of chemical energy from light energy, vary in size, toxicity, cell wall thickness, mobility and chemical composition. As a result, these organisms can be isolated in fisheries effluents, rivers, springs and wastewater facilities. The macronutrients and micronutrients that define the nutritional quality of water are relevant operational factors that determine the microalgae species that are able to predominate in a culture. Moreover, changes in microalgae predominance can be induced by changes in the growth medium produced by the own predominant species (Sanchis-Perucho et al. 2018). Microalgae can be grown discontinuously throughout the year from photobioreactors on an industrial scale (Guschina and Harwood 2006, Graef et al. 2009).

The success and economic viability of a microalgae-based biofuel industry will depend on a number of factors, including the selection of resistant strains that exhibit exceptional growth rates, lipid profiles suitable for biodiesel production and tolerance to a wide range of environmental parameters (Griffiths and Harrison 2009). However, so far, the identification of microalgae with potential for the production of biofuels is an issue still in formation, since there are many unexplored geographical areas and these may be important reservoirs of microalgae with potential for biodiesel production. For the above, it is of importance to isolate, identify and characterise microalgae. Such is the case for the state Chiapas that counts on an extensive hydrography with diverse climatic characteristics. Most likely, algae with key lipid content can be identified for biodiesel production and high biomass productivity. However, many physicochemical parameters are different amongst the strains and require their characterization and optimization individually. The identification and characterization of native microalgae from the state of Chiapas allows extending the biotechnological and energetic vision in the use of these organisms as a mechanism to take advantage of the biological diversitywhich Mexico has. For this purpose, the present research work had an objective to evaluate the population dynamics of microalgae isolated from different hydrographic areas of the state of Chiapas, Mexico and to identify morphotypes with potential for the production of lipids destined to the generation of biodiesel.

## Material and methods

### Collection of samples

Water samples used to isolate microalgae were collected aseptically from sites that appeared to contain algal bloom. About eight different water samples were collected from different locations in Chiapas, Mexico. Three samples of water of 1 litre per area were obtained from 6 rivers, 1 spring water and 1 filtering gallery of wastewater, each sample area was evaluated in triplicate, transported in coolers with 1 litre flasks per sample; the locations are presented in Table 1.

### Physical and chemical analyses of water samples

The physical and chemical properties of the water samples were determined for soluble chemical analysis, each sample being filtered through 0.2 µm syringe filters and stored at −20°C for subsequent analysis. Nitrate, nitrite and phosphate were measured using a Metrohm 850 Professional Ion Chromatograph (IC) (Metrohm Inc., Switzerland) with a Metrosep A Supp 5-250 anion column (Metrohm Inc., Switzerland). For anion analysis, 3.2 mM Na_2_CO_3_, with 1.0 mM NaHCO_3_ eluent were pumped at 2.6 ml/min, with a 100 mM HNO_3_ suppressor solution and using a 20 µl sample loop. The determinations were corroborated by the Hach kits (Hach, Loveland, CO, USA), per standard methods 10127, 8039 and 8507 for the determination total phosphorus, nitrates and nitrites, respectively. Ammonium was measured using Hach kits (Hach, Loveland, CO, USA), as per standard methods 8155. pH was measured using a Fisher-Scientific probe (Ben et al. 2008, Cho et al. 2017, Colombo et al. 2017, Stauch-White et al. 2017)

### Analysis of minerals in the water samples with the Atomic Absorption Spectrometer

Sample preparation consisted only of acidifying each water with 1% HNO3 (v/v) and adding 0.1% lanthanum chloride as a releasing reagent for calcium (Ca) and magnesium (Mg) and as an ionization suppressant for sodium (Na) and potassium (K). All analyses were carried out with the PerkinElmer PinAAcle 500 flame atomic absorption (AA) spectrometer. Measurements were made at a wavelength of 422.67, 324.75, 248.33, 285.21, 766.49, 589.00 and 213.86 nm for Ca, Cu, Fe, Mg, K, Na and Zn, respectively and a slit setting of 0.7 for Ca, Cu, Mg, K and Zn and 0.2 for Fe and Na. The visible range was used and the source current was set at 14 ma. A scale setting of 1 and an air flow rate of 2.5 l/min were employed. Before the start of each series of analyses, the gas (acetylene) flow rate was adjusted to give maximum absorbance while aspirating a standard solution. This value was usually 10 l/min. The aspiration rate was checked by using a stopwatch and graduated cylinder. Plugging of the aspirator was not excessive and, when it occurred, was rectified by aspirating 1:1 hydrochloric acid for 1 minute (Marguí et al. 2010; Dias Peronico and Raposo 2016).

### Isolation, purification and identification of microalgae

For the isolation process, 10 ml of water sample was transferred to a 500 ml conical flask containing 250 ml of sterilized BG11 medium and CHU medium (Wang et al. 2003). The flasks were incubated on a rotary orbital shaker at 150 rpm under continuous illumination using white fluorescent light at intensities of 3000 Lux for three weeks. Every two days, the flasks were examined for algal growth using an optical microscope. Subcultures were made by inoculating 50 ml of culture solution on to Petri plates containing the same isolation media solidified with 1.5% (w/v) of bacteriological agar. The purity of the culture was confirmed by repeated plating and also by repeated observation under a microscope. The obtained isolates were identified microscopically according to Wehr and Sheath (2003).

All microalgae species were morphologically validated; the latin scientific name and class were confirmed in the database of AlgaeBase” (http://www.algaebase.org) and database of NCBI (https://www.ncbi.nlm.nih.gov/pubmed).

### Determination of the microalgal biomass in BG11 and CHU medium

The dry cell weight (DCW) of microalgae biomass was also obtained by filtering 50 ml of aliquots of culture BG11 and CHU through a cellulose acetate membrane filter (0.45 µm pore size, 47 mm in diameter). Each loaded filter was dried at 105°C until the stability of weight was reached. The dry weight of the blank filter was subtracted from that of the loaded filter to obtain the microalgae dry cell weight (Wang et al. 2009).

### Determination of Size Frequency of Algal Cells

After treatment, aliquots were collected in 50 ml vials and then analysed to determine the size frequency of algal cells per ml of suspension using a FlowCam (Fluid imaging Technologies). The sizes were expressed as equivalent length (EL), according to the shape of the microalgae identified. FlowCam is a continuous imaging flow cytometer designed to characterize particles that pass through a flow chamber. The FlowCam captures digital images of particles in a fluid stream using laser light detection, enabling the measurement of many cell parameters, such as length (Sieracki et al. 1998). To begin, the FlowCam, including the integrated computer and laser, was turned on. Then the FlowCam software programme (vs 20x programme) was opened. The focus on the camera was adjusted to ensure clear images and the flow cell checked and cleared of any bubbles or debris. The autoimage mode was set for 2 minutes. A video camera or framegrabber captures an image of each object that passes through the field of view on a 20x objective microscope lens. The digitized images are then collected and stored in the computer where they can be analysed with FlowCam software.

### Statistical Analysis

The statistical software used was the STATGRAPHICS PLUS (1999) for windows. For the first experiment, prior to statistical analysis, data were assessed for equality of variance and normality. The proportion of the size range of algae concentrate between 1 and 10 μm was transformed to Arcsin square root to improve the homogeneity of variance assumption (Patel et al. 2015). For statistical analyses, one-way ANOVA was used at p<0.05 level of significance.

## Data resources

All microalgae species were morphologically validated; the latin scientific name and class were confirmed in the database of AlgaeBase (http://www.algaebase.org) and database of NCBI (https://www.ncbi.nlm.nih.gov/pubmed).

## Results

Once the samples were taken, they were evaluated to identify the presence of microalgae, in such a way that microalgae and cyanobacteria that remain associated in consortiums were identified.

Physical and chemical analyses of water samples

In the physicochemical characterization of the hydrographic areas of the state of Chiapas, it was observed that the Santo Domingo River presented the highest concentrations of nitrates, nitrites and ammonium with 0.03, 0.006 and 0.08 mg/l, respectively. However, the filtration gallery of the wastewater “La Chacona” presented the lowest concentrations of nitrates, nitrites, ammonia and total phosphorus with 0.01, 0.002, <0.01 and 0.08 mg/l respectively (Table 2).

The Santo Domingo River presented the highest concentrations of nitrogen compared to the other hydrographic areas. This is related to the relative abundance in the identification of morphotypes, since it was the area with the highest number of morphotypes identified (29%).

Analysis of minerals in the water samples with an Atomic Absorption Spectrometer

In the evaluation of minerals, the 8 hydrographic zones of the analyzed Chiapas state showed concentrations lower than 0.2 mg/l of microelements such as copper, zinc, iron and manganese. However, the Santo Domingo river showed the highest concentrations of sodium, potassium, magnesium and calcium with 62.93, 5.46, 34.52 and 48.78 mg/l, respectively. On the other hand, the Pijijiapan river showed the lowest concentrations of sodium, potassium, magnesium and calcium with 8.87, 0.05, 0.81 and 6.59 mg/l, respectively (Table 3).

Isolation, purification, identification and determination of size of microalgae

**Figure 1.**
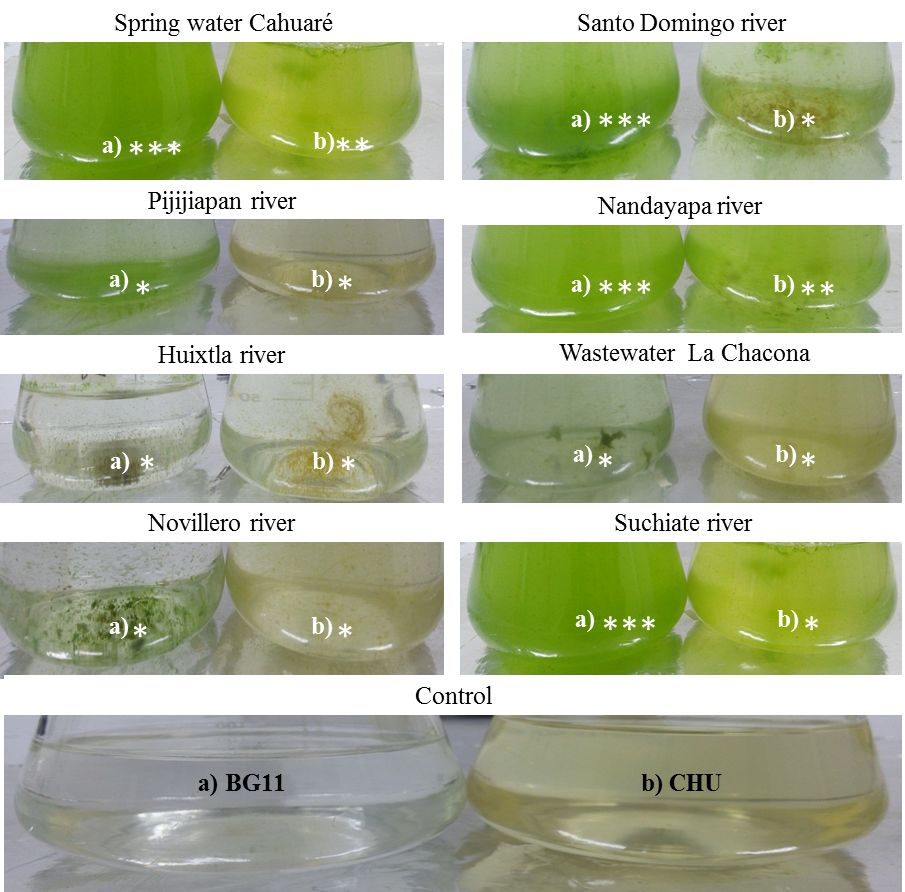
Visual evaluation of the efficiency for isolation in culture media BG11 (a) and CHU (b) from the production of microalgal biomass; Level of concentration is observed with the number of asterisks: ***High concentration; **Average concentration;* Low Concentration.

In our study, more than twenty-one isolates were isolated from the collected water samples, but only thirteen axenic microalgae isolates were selected and sub-cultured on slants on its specific isolation media (BG11) and kept in a refrigerator for further investigation due to their purity. The isolation of microalgae was developed with the use of BG11 and CHU culture media from which, after a period of 90 days with a 12/12 photoperiod, it was demonstrated that the most efficient means for the isolation of microalgae was the medium BG11 with an 80.53% effectiveness. The CHU medium was, however, efficient at 19.46%, showing a statistically significant difference with α= 0.05 (Fig. 1 and Fig. 5).

In Fig. 1, it is observed that, in Suchiate river and Santo Domingo rivers, they presented greater growth of microalgal biomass in BG11 medium at 90 days after inoculation.

**Figure 2.**
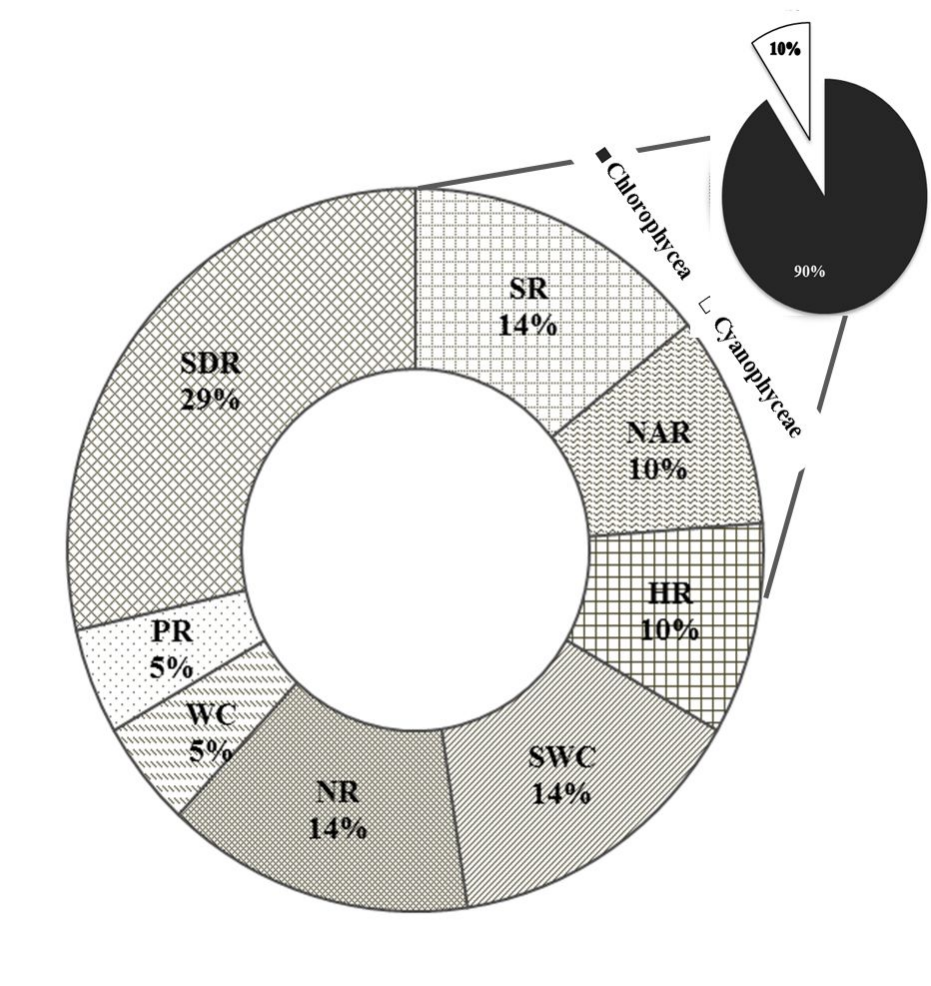
Relative abundance of the morphotypes and classes of microalgae identified in the different hydrographic zones evaluated in the state of Chiapas. SR: Suchiate river; NAR: Nandayapa river; HR: Huixtla river; SWC: Spring water Cahuaré; NR: Novillero river; WC: Wastewaters "La chacona"; PR: Pijijiapan river; SDR: Santo Domingo river.

Of the water samples analyed, 13 microalgae morphotypes were isolated. Of the microalgal consortia identified, 90.47% of a total of 21 microorganisms are composed of microalgae belonging to the class Chlorophyceae and9.52% correspond to the class Cyanophyceae, as shown in Fig. 2.

It is important to mention that the areas with the highest microalgae specific richness were the Santo Domingo River of the municipality of Chiapa de Corzo with 6 identified morphotypes corresponding to 28.57%, followed by the river Suchiate of the municipality of Suchiate with 3 identified morphotypes corresponding to 14.28% of the relative abundance (Fig. 2).

**Figure 3.**
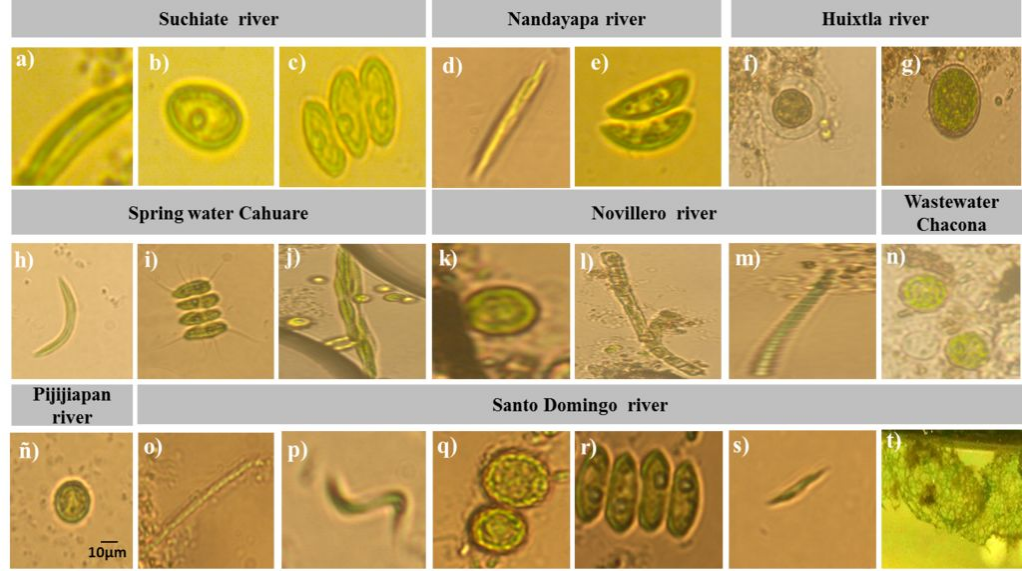
Microalgae identified in the different hydrographic zones of the state of Chiapas using a FlowCam coupled to a microscope (100x). **Suchiate river**: a) *Monoraphidium contortum*; b) *Neospongiococcum gelatinosum*; c) *Desmodesmus serratus*; **Nandayapa river**: d) *Raphidonema nivale*; e) *Nephrocytium lunatum*; **Huixtla river**: f) *Asterococcus superbus*; g) *Chlorococcum echinozygotum*; **Spring water Cahuaré**: h) *Monoraphidium contortum*; i) *Scenedesmus quadricauda*; j) *Monoraphidium griffithii*; **Novillero river**: k) *Chlorococcum echinozygotum*; l) *Leptolyngbya* sp.; m) *Microspora floccosa*; **Wastewaters "La chacona**": n) *Asterococcus superbus*; **Pijijiapan river**: ñ) *Chlorococcum echinozygotum*; **Santo Domingo river**: o) *Oscillatoria brevis*; p) *Monoraphidium contortum*; q) *Chlorococcum echinozygotum*; r) *Desmodesmus serratus*; s) *Closteriopsis acicularis*; t) *Hydrodictyon reticulatum*.

According to morphological examination under a microscope based on cell shapes, fourteen microalgal isolates were identified as *Monoraphidium contortum*, *Neospongiococcum gelatinosum*, *Desmodesmus serratus*, *Raphidonema nivale*, *Nephrocytium lunatum*, *Asterococcus superbus*, *Chlorococcum echinozygotum*, *Scenedesmus quadricauda*, *Monoraphidium griffithii*, *Leptolyngbya* sp., *Microspora floccosa*, *Oscillatoria brevis*, *Closteriopsis acicularis* and *Hydrodictyon reticulatum.* The aforementioned morphotypes are shown in Fig. 3.

**Figure 4.**
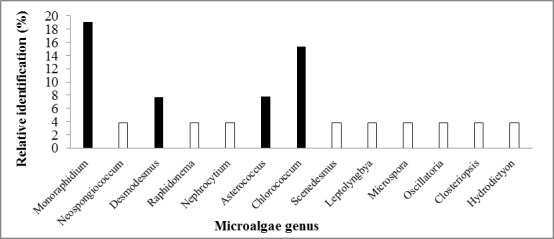
Relative abundance of the different genera of microalgae identified in hydrographic zones in the state of Chiapas, Mexico.

As can be seen in Fig. 3, there is a great diversity of microalgae morphotypes with specific characteristics; however, it is important to mention that 13 different genera were identified, of which four had greater abundance, these being *Monoraphidium* and *Chlorococcum* with 18% and 16%, respectively and the genera *Asterococcus* and *Desmodesmus* with 7.69%, as observed in Fig. 4.

Of the 14 identified morphotypes, the measurements were between 10 μm to 150 μm in length. The identified morphotypes of larger size were *Monoraphidium griffithii* and *Microspora floccosa* with 50 and 150 μm, respectively, both corresponding to the class Chlorophyceae (Table 4).

The algae were identified through the morphological analysis based on the general features of the freshwater algae that occur most frequently and are described by Bellinger and Sigee (2010)Ettl and Schlösser (1992) and Wehr and Sheath (2003). Morphological observation was performed using light-field microscopy, according to the size and comparison with general records of external taxonomic databases such as AlgaeBase” (http://www.algaebase.org) and database of NCBI (https://www.ncbi.nlm.nih.gov/pubmed), the authors (Eibl et al. 2014 mentioning that these data are relevant and important for the identification of new morphotypes (Table 4).

Determination of the microalgal biomass in BG11 and CHU medium

**Figure 5.**
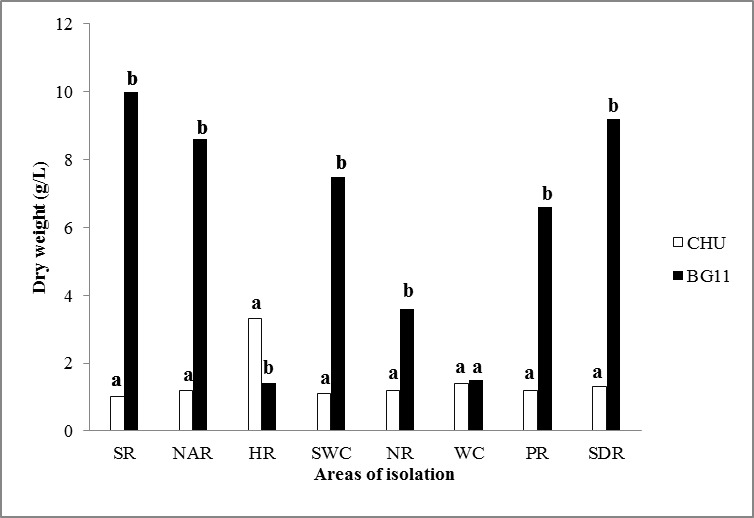
Evaluation of microalgal biomass production in BG11 and CHU medium after a period of 90 days with a 12/12 photoperiod for the different hydrographical areas of the state of Chiapas: SR: Suchiate river; NAR: Nandayapa river; HR: Huixtla river; SWC: Spring water Cahuaré; NR: Novillero river; WC: Wastewaters "La chacona"; PR: Pijijiapan river; SDR: Santo Domingo river. Bars are + one standard deviation. A means values of three replicates. The means followed by the same letter are not significantly different (P-value <0.05).

After a period of 90 days with a 12/12 photoperiod, the evaluation of the BG11 and CHU medium was developed to determine the effectiveness of both media in the production of microalgal biomass that allowed the identification of the existing morphotypes of microalgae. For that effect, greater microalgal biomass production was demonstrated in the BG11 medium from 1.4 to 10 g while the CHU medium showed a production of 1 to 1.4 g of microalgal biomass, so that a significant statistical difference between the two media was observed. Therefore, the BG11 medium is the better medium for the isolation and biomass production (Fig. 5).

It was observed that, in the Suchiate river and the Santo Domingo river, greater growth of microalgal biomass was demonstrated in the BG11 medium with 10 g and 9.2 g respectively after 90 days of inoculation (Fig. 5).

## Discussion

In the present research work, 14 morphotypes were identified according to their qualitative and quantitative morphological characteristics (Table 4) such as presented by Romero et al. 2012; they developed the taxonomic identification of marine actinomycetes by means of a morphological analysis in the first instance and the screening was carried out through a rapid selection of strains by visual inspection of the colour. Wu et al. 2013 also performed the Light Microscopic analysis of the samples of the isolated microalgae, allowing the morphological identification of the genus *Cosmarium*.

The Santo Domingo River was the area with the highest number of morphotypes identified, the results showing the importance of nitrogen as explained by Yuan et al. (2014) who established that nitrogen is a fundamental element for the formation of proteins and nucleic acids, these being an integral part of essential molecules such as ATP, the energy carrier in cells. Breuer et al. (2012) also mention that the nitrogen supplied to the microalgae in the culture medium allows it to produce proteins and nucleotides that are part of the biomass. When nitrates are supplied to the culture medium, microalgae denitrify nitrate (NO_3_-) to inorganic nitrogen (N_2_); once the inorganic nitrogen is obtained, it enters as NH_2_ into the microalgal proteins. It is important to mention that another factor favouring the richness of morphotypes identified in the Santo Domingo River is associated with an adequate concentration of ammonium as was established by Ayre et al. (2017). They evaluated if microalgae species were capable of growing on undilutedanaerobic digestate of piggery effluent (ADPE). Thus outdoor growth of the mixed culture, using raceway ponds, showed the potential for up to 0.0637 ± 12.1 g N-NH_4_^+^ l^−1^ ammonium removal from the ADPE.

Another important element for the production of microalgae is phosphorus because it is a macroelement that is found in smaller proportions than the rest in the biomass and are used by microalgae in the synthesis of enzymes, lipids and nucleic acids (Xin et al. 2010,Tan et al. 2016). Thus, the correct relationship between N and P is very important to guarantee the production of microalgal biomass (Pereira et al. 2016), as demonstrated by Myint (2014) who mention that it is convenient to handle an optimum proportion ratio of nitrogen and phosphorus (N:P) for the growth of microalgae (7:1). According to the aforementioned, in Table 2, it is observed that the ratio N:P is 3:1 for the Santo Domingo river, so it is the hydrographic zone with the best N:P ratio close to the optimum ratio for the production of microalgal biomass.

According to the results obtained in Table 3, the Santo Domingo River showed high concentrations of sodium, potassium, magnesium and calcium, which allowed the relative abundance of the identified morphotypes as mentioned by Hussain et al. (2017) who established that the nutrients present in the culture medium maintain osmotic pressure and electrolyte balance. Some nutrients are part of the biomass of proteins, especially the Mg ion that is located in the centre of the porphyrin nucleus of the chlorophylls which are important actors in the process of photosynthesis. Thus, microelements such as Mn, Cu, Co and Zn act as co-factors for enzymes, producing vitamins and maintaining the cell wall. Their concentration in the culture medium are so low that their excess can be toxic to microalgae, given the conditions of the different hydrographic areas of the state of Chiapas which have been evaluated with low concentration of microelements, it was possible to identify microalgae (Valverde-Pérez et al. 2015).

The genera identified are part of the chlorophyce class whose abundance was evaluated by Blokker et al. (1998) who demonstrated that the cellular walls of fresh or residual freshwater microalgae are composed of highly resistant aliphatic biopolymers, not hydrolyzable by thermal and chemical degradations. Biopolymers are composed of long chains of fatty acids that vary in length (from 30 to 34 carbon atoms). These intermolecular monomers linked by an ester group form linear chains that resist the unsaturations. The nature of these polyether algae makes them highly resistant todegradation, so they are conserved in rivers with residual flow or high pollutant load flows (Cardoso et al. 2012), characteristics that predominate in the rivers of the state of Chiapas, analyzed in the present study.

Therefore, in the present research work, it is demonstrated that the nutrient richness of the different hydrographic areas allows the identification of the population dynamics of the microalgae in specific taxonomic groups, as was demonstrated by Domenighini and Giordano (2009). They showed that it is possible to accurately identify the species according to the nutritional status of their home environment (for example, N source) in addition to the evaluation of biodiversity in natural phytoplankton samples, that allows control of the water quality of the natural environment.

Garrido-Pérez et al. (2003) also carried out the analysis of the nutritional status of water (final oligotrophy of eutrophy) as a bioassay for the identification of marine microalgae, generating a relationship between the biological quality of water and morphotypes and demonstrating that this nutritional richness comprises a rank specific taxonomy.

Of the 13 species identified in the present study, 5 have been studied in order to identify fatty acids for the production of biodiesel, so that in Table 5, it can be seen that the lipid content in the identified microalgae of hydrographic areas of the state of Chiapas can be from 2% to 90%, according to studies developed by Bogen et al. (2013), Rajab Aljuboori et al. (2016), Bonnefond et al. (2016) and Sattar Memon et al. (2016), Chlorococcum and Monoraphidium being the most abundant in this study, with important contents of lipids reported (Table 5).

Observing the potential of *Microspora floccosa* to produce up to 90% of lipids, we can generate a relationship between the nutrients present in the medium from which they were isolated. Thus we observed that this morphotype comes from the Novillero river, which is characterized by low concentrations of nitrate, nitrite and ammonium with 0.02, 0.005 and <0.001 mg/l, respectively and the lowest concentration of sodium with 9.07 mg/l, so that this relationship benefits the synthesis of lipids in favour of the production of bioenergetics as demonstrated by Liang et al. (2017). It was suggested that low salinities and N starvation are considered efficient ways to stimulate lipid accumulation in D- tertiolecta. Therefore *Microspora floccosa* is a microalga with potential for the production of biodiesel (Table 5).

In the present study, the BG11 culture medium showed higher efficiency than the CHU medium in the production of microalgal biomass, the phenomenon observed being demonstrated by Ambrosio et al. (2017), when evaluating the metabolic engineering in the absorption and assimilation of nitrogen and carbon in plants and microalgae, to include sources of C and N, resulting in a strong proliferation of microalgae. In order to achieve optimal growth of microalgae, it is important to determine the amount of different nutrients to be added in the culture medium.

The most important nutrients are those that represent sources of carbon and nitrogen in the culture medium (Bilanovic et al. 2016), so the BG11 medium has higher concentrations of N and C in 6 of its chemical compounds while the CHU medium includes these elements in 4 of its chemical molecules (Patel et al. 2015).

At the metabolism level, carbon is vital for microalgae as an energy generator from the Calvin cycle (Soreanu et al. 2017). On the other hand, the nitrogen supplied to the microalgae in the culture medium allows it to produce proteins that are part of the biomass (Breuer et al. 2012). As shown by Pancha et al. (2014), their results revealed that nitrogen limitation and sequential nitrogen starvation conditions significantly reduced the photosynthetic activity and thus the production of microalgal biomass of *Scenedesmus* sp.

It is important to mention that the identification of new morphotypes in different hydrographic space generates an area of scientific research to identify the biotechnological potential of microalgae associated with the high generation of lipids, proteins, pigments or as environmentally beneficial microorganisms involved in bioremediation processes. Thus the new isolations also allow the observation of the taxonomic evolution of microalgae classes, demonstrating the conservation, loss and/or appearance of biomolecules (Bogen et al. 2013a, Serive et al. 2017, Bogen et al. 2013b).

## Conclusions

Fourteen morphotypes were isolated and identified according to the morphological classification of Wehr and Sheath (2003) and similarity validation according to the Algabase algae bank and the NCBI database (Table 4, Fig. 3) The microalgae were isolated from different hydrographic areas of the state of Chiapas, of which 90% belong to the class Chlorophyceae and 10% correspond to Cyanophyceae (Fig. 2). It is important to mention that the area with the highest microalgae specific richness was the Santo Domingo River of the municipality of Chiapa de Corzo with 6 identified morphotypes corresponding to 28.57% of the total identified, being associated with its high nutritional wealth and demonstrating highest concentrations of nitrates, nitrites and ammonium with 0.03, 0.006 and 0.08 mg/l, respectively (Table 2, Table 3). Finally, we identified 5 species with potential for the production of biodiesel with a lipid content of 2 to 90% according to literature (Table 5).

## Figures and Tables

**Table 1.** Distribution of sample collection of the different hydrographic zones of the state of Chiapas, Mexico.

**N.**	**River**	**Town**	**Geographical coordinates**
1	Nandayapa	Acala	16°33’12”N; 92°48’25”W
2	Huixtla	Huixtla	15°08′00″N; 92°28′00″W
3	Novillero	Tonalá	16°05′22″N; 93°45′05″W
4	Sto Domingo	Chiapa de Corzo	16°45′11″N; 93°06′56″W
5	Suchiate	Suchiate	14°41′00″N; 92°09′00″W
6	Pijijiapan	Pijijiapan	15°41′12″N; 93°12′33″W
7	Spring water Cahuaré	Chiapa de Corzo	16°42′30″N; 93°01′01″W
8	Filter gallery of wastewater “La Chacona”	Tuxtla Gutiérrez	16°45′11″N; 93°06′56″W

**Table 2.** Physicochemical characterization of the water of the different hydrographic zones of the state of Chiapas, Mexico.

**Sampling areas**	**Nitrates (mg/l)**	**Nitrites (mg/l)**	**Ammonium (mg/l)**	**Total phosphorus (mg/l)**
Suchiate river	*0,02ª + 0.04	0,003^ab^ + 0.01	0,04^b^ + 0.01	0,18ª + 0.02
Nandayapa river	0,02ª + 0.01^**^	0,003^ab^ + 0.01	< 0,01^c^ + 0.00	< 0,02^d^ + 0.00
Huixtla river	0,02ª + 0.02	0,005^ab^ + 0.02	< 0,01^c^ + 0.00	0,12^b^ + 0.01
Spring water Cahuaré	0,03ª + 0.04	0,004^ab^ +0.00	< 0,01^c^ + 0.00	0,1^bc^ + 0.01
Novillero river	0,02ª + 0.01	0,005^a^ + 0.01	< 0,01^c^ + 0.00	0,1^bc^ + 0.01
Wastewaters "La chacona"	0,01ª + 0.00	0,002^b^ + 0,00	< 0,01^c^ + 0.00	0,08^c^ + 0.02
Pijijiapan river	0,02^a^ + 0.01	0,002^b^ + 0.00	0,04^b^ + 0.01	0,04^d^ + 0.02
Santo Domingo river	0,03^a^ + 0.02	0,006^a^ + 0.00	0,08^a^ + 0.02	0,03^d^ + 0.01

* Mean values of three replicates;

** Means (±standard error) within each column, with no common superscript, differ significantly at P <0.05.

**Table 3.** Evaluation of the concentration of macroelements and microelements of water samples from the different hydrographic zones of the state of Chiapas, Mexico.

**Sampling areas**	**Microelements**				**Macroelements**			
	**Copper** **(mg/l)**	**Zinc** **(mg/l)**	**Iron** **(mg/l)**	**Manganese** **(mg/l)**	**Sodium** **(mg/l)**	**Potassium** **(mg/l)**	**Magnesium** **(mg/l)**	**Calcium** **(mg/l)**
Suchiate river	< 0.2^a*^ + 0.01	< 0.2^a^ + 0.01	< 0.2^a^ + 0.00	< 0.2^a^ + 0.01	17,09^d^ + 0.11	2,77^ab^ + 0.01	5,89^d^ + 0.02	14,36^c^ + 0.11
Nandayapa river	< 0.2^a^ + 0.01	< 0.2^a^ + 0.01	< 0.2^a^ + 0.01	< 0.2^a^ + 0.11	57,87^b^ + 0.12	4,13^a^ + 0.12	21,45^b^ + 0.16	51,06^a^ + 0.15
Huixtla river	< 0.2^a^ + 0.03^**^	< 0.2^a^ + 0.00	< 0.2^a^ + 0.01	< 0.2^a^ + 0.00	23,71^c^ + 0.03	2,76^ab^ + 0.11	13,74^c^ + 0.04	13,78^c^ + 0.12
Spring water Cahuaré	< 0.2^a^ + 0.02	< 0.2^a^ + 0.01	< 0.2^a^ + 0.02	< 0.2^a^ + 0.02	17,06^d^ + 0.17	2,41^ab^ + 0.10	14,97^c^ + 0.09	45,62^a^ + 0.13
Novillero river	< 0.2^a^ + 0.01	< 0.2^a^ + 0.02	< 0.2^a^ + 0.01	< 0.2^a^ + 0.01	9,07^e^ + 0.14	0,92^b^ + 0.01	1,63^e^ + 0.02	15,06^c^ + 0.09
Wastewaters "La chacona"	< 0.2^a^ + 0.01	< 0.2^a^ + 0.01	< 0.2^a^ + 0.00	< 0.2^a^ + 0.00	10,82^e^ + 0.08	1,69^b^ + 0.11	13,51^c^ + 0.11	40,50^b^ + 0.12
Pijijiapan river	< 0.2^a^ + 0.00	< 0.2^a^ + 0.03	< 0.2^a^ + 0.03	< 0.2^a^ + 0.02	8,87^e^ + 0.07	0,05^c^ + 0.09	0,81^e^ + 0.01	6,59^d^ + 0.09
Santo Domingo river	< 0.2^a^ + 0.01	< 0.2^a^ + 0.02	< 0.2^a^ + 0.00	< 0.2^a^ + 0.00	62,93^a^ + 0.14	5,46^a^ + 0.14	34,52^a^ + 0.12	48,78^a^ + 0.11

*Mean values of three replicates.

**Means (±standard error) within each column, with no common superscript, differ significantly at P <0.05.

**Table 4.** Analysis of the morphotypes identified in the hydrographic zones of the state of Chiapas according to the environment of isolation, class, size and morphological characteristics.

**N.**	**Name**	**General environment**	**Size (μm)**	**Characteristics**	**References**
	** CLOROPHYCEAE **				
1	*Monoraphidium contortum* ID: 307511	Sweet water	40	It has a fusiform body, narrow towards the extremities, with a sharp point, is sigmoid and also contains two parietal chloroplasts	Fawley et al. 2006
2	*Neospongiococcum gelatinosum* ID: 1158268	Land	21	Multinucleate species, formed by a rigid wall, is characterized by being individualistic and staying inside a gelatinous sphere	Guiry 2018
3	*Desmodesmus serratus* ID: 91204	Sweet water	26	Cenobios 2-4-8 linear cells. Ovoid cells with 1-4 teeth at the ends that are rounded or truncated, external convex walls	Guiry and Guiry 2018
4	*Raphidonema nivale* ID: 155715	Freshwater/terrestrial species	20	A filamentous structure, forming a green, non-greenish cenocito approximately 0.5 μm thick with a polar tip	Fott 1970
5	*Nephrocytium lunatum* ID: 1662585	Sweet water	31	Colonies of 2-4 cells, embedded within a gelatinous sheath; cell body in the shape of a crescent, contains a single plate as a chloroplast with a pyrenoid	Murphy 2003
6	*Asterococcus superbus* ID: 269637	Sweet water	25	The body is spherical and ellipsoidal. It has a smooth and transparent cell wall containing a single star-shaped chloroplast, a single nucleus and two contractile vacuoles	Korshikov 1953
7	*Chlorococcum echinozygotum* ID: 48000	A terrestrial species	15	Almost spherical cells. Membrane not thickened in anterior papilla. Chloroplast with a large pyrenoid. Large eye patch.	David and Brian 2011
8	*Scenedesmus quadricauda* ID: 3089	Sweet water	15	Colonial individual, consisting of 4, 8 or 12 cells. The central cells are elongated and without appendages, the terminals bulge in the centre and present two spines that project towards the outside.	Day and Wiskich 1995
9	*Monoraphidium griffithii* ID: 307514	Sweet water	50	Spindle cells, straight, more than 12 times longer than wide (50-72 x 1.5-4.5 μm), attenuated towards the ends and terminated at a short point. Clearly constricted parietal chloroplast in the centre and without pyrene	Peixoto Ramos et al. 2012
10	*Hydrodictyon reticulatum* ID: 3107	Fresh and wastewater	30	It has a transparent net shape with a green sack-like shape with thousands of cylindrical oblong-oval cells, with thick and angled walls	Buchheim et al. 2005
11	*Microspora floccosa* ID: 1603044	Sweet water	150	Green H-shaped row, formed by cylindrical cells, the ends of the filaments have an adhesive disc with which they are fixed, chloroplasts do not enclose pyrenoids	Hu 2006
12	*Closteriopsis acicularis* ID: 82138	Sweet water	10	Single-celled stems elongated and pointed tips, central or parietal chloroplasts in the form of band, formed by multiple pyrenoids of 2 to 14.	Krienitz et al. 2004
	** CYANOPHYCEAE **				
13	*Oscillatoria brevis* ID: 177969	Sweet water	70	Each filament consists of trichomes that are composed of rows of cells, formed by fragments called hormogonias. The tip of the tricoma oscillates like a pendulum, of green brown colour.	Anagnostidis and Komárek 1998
14	*Leptolyngbya* sp. ID: 47254	Fresh and wastewater	62	Long, solitary or coiled filaments in groups and thin mats formed by generally colourless facultative pods attached to or slightly distant from the trichomes which are composed of rounded apical cells	Komárek 2005

**Table 5.** Lipid content of five microalgae with potential for biodiesel production identified in the hydrographic zones of the state of Chiapas, Mexico.

**Species of microalgae**	**Lipid content (%)**	**References**
*Monoraphidium contortum*	2-20	Bogen et al. 2013
*Chlorococcum echinozygotum*	10-43	Graef et al. 2009
*Scenedesmus quadricauda*	11-55	Rajab Aljuboori et al. 2016
*Microspora floccosa*	04-90	Sattar Memon et al. 2016
*Dunaliella salina*	09-47	Bonnefond et al. 2016

## Hosting institution

The institution hosting were 3IER, UPCH, for financial support.
